# Supplementary material for: Efficacy and Safety of Glycosidic Enzymes for Improved Gene Delivery to the Retina following Intravitreal Injection in Mice
Source: Mol Ther Methods Clin Dev. 2017 Dec 22;9:192–202. doi: 10.1016/j.omtm.2017.12.002 (PMC5948313; doi:10.1016/j.omtm.2017.12.002)
Supplement: Document S1. Figures S1 and S2 [file mmc1.pdf]

OMTM, Volume *9*

Supplemental Information

Efficacy and Safety of Glycosidic Enzymes for Improved Gene Delivery to the Retina following Intravitreal Injection in Mice

Jasmina Cehajic-Kapetanovic, Nina Milosavljevic, Robert A. Bedford, Robert J. Lucas, and Paul N. Bishop

Supplemental Material

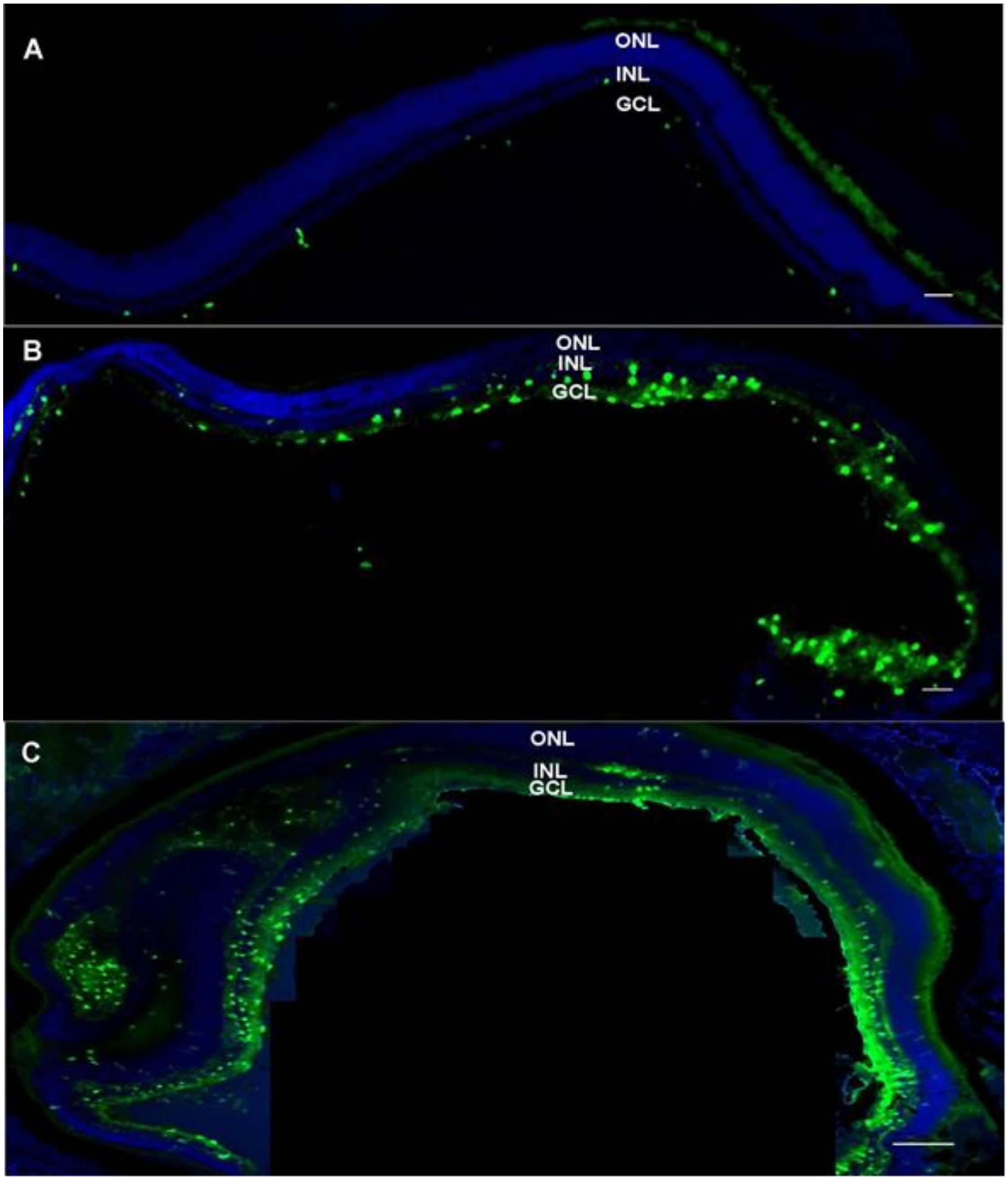

Supplemental Figure 1.

Transgene expression in *wild-type* retina using low dose AAV2-CAG-GFP in conjunction with glycosidic enzymes

(A-C) Exemplar images of transverse sections through a *wild-type* mouse retina >6 weeks after intravitreal delivery of AAV2-CAG-GFP ($2x10^8$ gc/eye) alone (A) or in conjunction with heparinase III (B) and hyaluronan lyase (C). Unamplified expression of GFP in cells of the ganglion cell layer (GCL) and inner nuclear layer (INL) is shown in green. Nuclei are counterstained with DAPI (blue). Calibration bar = 50μm.

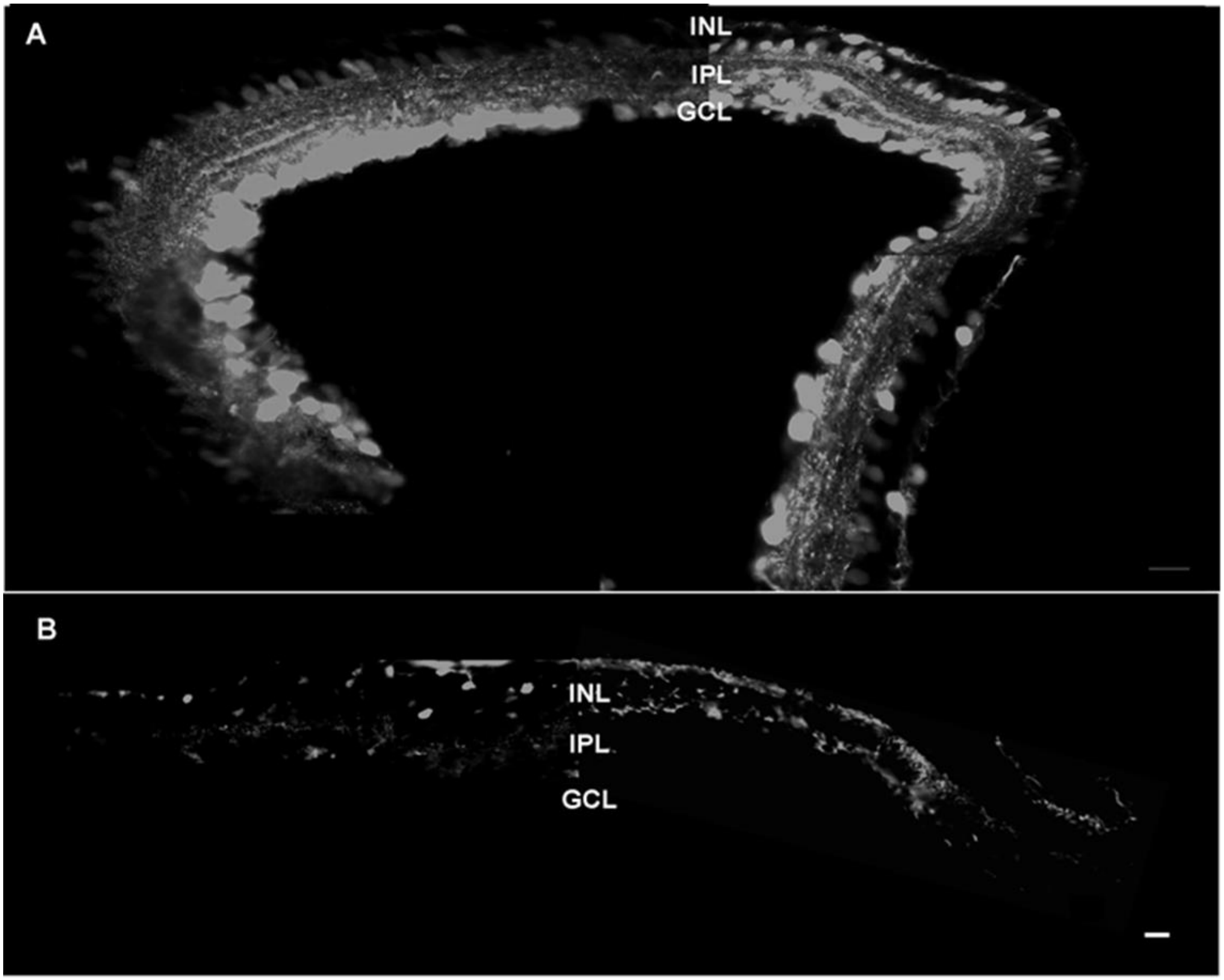

Supplemental Figure 2

Transgene expression in *rd*[1] retina using high dose AAV2-GFP in conjunction with glycosidic enzymes

(A, B) Montage of several high magnification images from an exemplar transverse section through an *rd*[1] mouse retina >6 weeks after intravitreal delivery of (A) AAV2- CAG-GFP (3 x10^{10} gc/eye) or (B) AAV2-grm6-GFP (3x10^{10} gc/eye) in conjunction with heparinase III and hyaluronan lyase combined. Expression of enhanced green fluorescent protein in cells of the ganglion cell layer (GCL) and inner nuclear layer (INL) is seen in (A) and in cells of the INL only in (B). Calibration bar = 50µm.
