# Supplementary material for: Efficacy and Safety of Glycosidic Enzymes for Improved Gene Delivery to the Retina following Intravitreal Injection in Mice
Source: Mol Ther Methods Clin Dev. 2017 Dec 22;9:192–202. doi: 10.1016/j.omtm.2017.12.002 (PMC5948313; doi:10.1016/j.omtm.2017.12.002)
Supplement: Document S2. Article plus Supplemental Information [file mmc2.pdf]

Molecular Therapy
Methods & Clinical Development
Original Article

Efficacy and Safety of Glycosidic Enzymes for Improved Gene Delivery to the Retina following Intravitreal Injection in Mice

Jasmina Cehajic-Kapetanovic,[1,2] Nina Milosavljevic,[1] Robert A. Bedford,[1] Robert J. Lucas,[1] and Paul N. Bishop[1,2]

[1]Faculty of Biology, Medicine and Health, University of Manchester, Oxford Road, Manchester M13 9PT, UK; [2]Manchester Royal Eye Hospital, Manchester University NHS Foundation Trust, Manchester Academic Health Sciences Centre, Manchester, UK

Viral gene delivery is showing great promise for treating retinal disease. Although subretinal vector delivery has mainly been used to date, intravitreal delivery has potential advantages if low retinal transduction efficiency can be overcome. To this end, we investigated the effects of co-injection of glycosaminoglycan-degrading enzymes, singly or in combination, with AAV2 as a method of increasing retinal transduction. Experiments using healthy mice demonstrated that these enzymes enhance retinal transduction. We found that heparinase III produced the greatest individual effect, and this was enhanced further by combination with hyaluronan lyase. In addition, this optimized AAV2-enzyme combination led to a marked improvement in transduction in retinas with advanced retinal degeneration compared with AAV2 alone. Safety studies measuring retinal function by flash electroretinography indicated that retinal function was unaffected in the acute period and at least 12 months after enzyme treatment, whereas pupillometry confirmed that retinal ganglion cell activity was unaffected. Retinal morphology was not altered by the enzyme injection. Collectively these data confirm the efficacy and safety of this intravitreal approach in enhancing retinal transduction efficiency by AAV in rodents. Translating this method into other species, such as non-human primates, or for clinical applications will have challenges and require further studies.

INTRODUCTION

Inherited retinal degenerations (retinal dystrophies) are a major cause of blindness, affecting approximately 1 in 2,500 people worldwide. In most forms, genetic mutations affect the cells in the outer retina—i.e., the photoreceptors and retinal pigment epithelium (RPE)—making these cells primary targets for emerging gene-based therapies. The landmark ocular gene therapy clinical trials for Leber congenital amaurosis 2 (LCA2), a rare form of inherited retinal degeneration,[1–5] have demonstrated the safety and efficacy of delivering therapeutic transgenes via an adeno-associated virus (AAV) vector to the RPE by subretinal injection. However, in LCA2, the retinal architecture can remain intact for many years,[6] in advanced retinal degeneration, the retina can become thin and fragile, making subretinal delivery of AAV vectors challenging and prone to complications.[5,7–10] An alternative approach is intravitreal injection, a technically less challenging procedure with a lower risk of complications and, therefore, a more broadly applicable approach compared with subretinal injection. However, reaching therapeutic levels of transduction in the retina from the vitreous presents challenges and has been a focus of a number of recent preclinical gene therapy studies.[11–21]

AAV-based vectors are currently being developed that are capable of transducing the outer retina in animal models following intravitreal injection using rational mutagenesis[13–20] or *in vivo*-directed evolution.[21–23] Rational mutagenesis manipulates viral capsids (surface-exposed tyrosine, threonine, and lysine residues) to decrease intracellular ubiquitination and proteosomal degradation of the vector, resulting in increased retinal transduction. Directed evolution selects AAV variants from combinatorial libraries with desirable cellular tropism *in vivo*. Thus, through multiple cycles of evolution, it enriches for AAV variants with specific cell tropism (e.g., the Sh10 variant for Müller cells)[21] or those capable of reaching the outer retina from the vitreous through altered receptor-binding properties such as the 7m8 variant.[23] These novel AAV variants have been shown to produce a more effective functional rescue of disease phenotype in animal models of retinal degeneration.[23,24]

An alternative strategy for increasing retinal transduction following intravitreal delivery is to tackle the physical barriers to vector penetration of the retina. Naturally occurring AAV serotypes produce limited inner retinal transduction and are ineffective in transducing the outer retina via intravitreal delivery because the vitreous, inner limiting membrane (ILM), retinal extracellular matrix (ECM), and cell surface proteoglycans form substantial barriers and binding sites that immobilize the AAV particles.[11,12,25] We demonstrated previously that enzymatic lysis of these barriers, using glycosidic enzymes, improved the depth and efficiency of vector penetration, leading to more

Received 5 August 2017; accepted 19 December 2017;
https://doi.org/10.1016/j.omtm.2017.12.002.

Correspondence: Robert J. Lucas, Faculty of Biology, Medicine and Health, AV Hill Building, University of Manchester, Oxford Road, Manchester M13 9PL, UK.
E-mail: robert.lucas@manchester.ac.uk
Correspondence: Paul N. Bishop, Faculty of Biology, Medicine and Health, AV Hill Building, University of Manchester, Oxford Road, Manchester M13 9PL, UK.
E-mail: paul.bishop@manchester.ac.uk

This is an open access article under the CC BY license (http://creativecommons.org/licenses/by/4.0/).

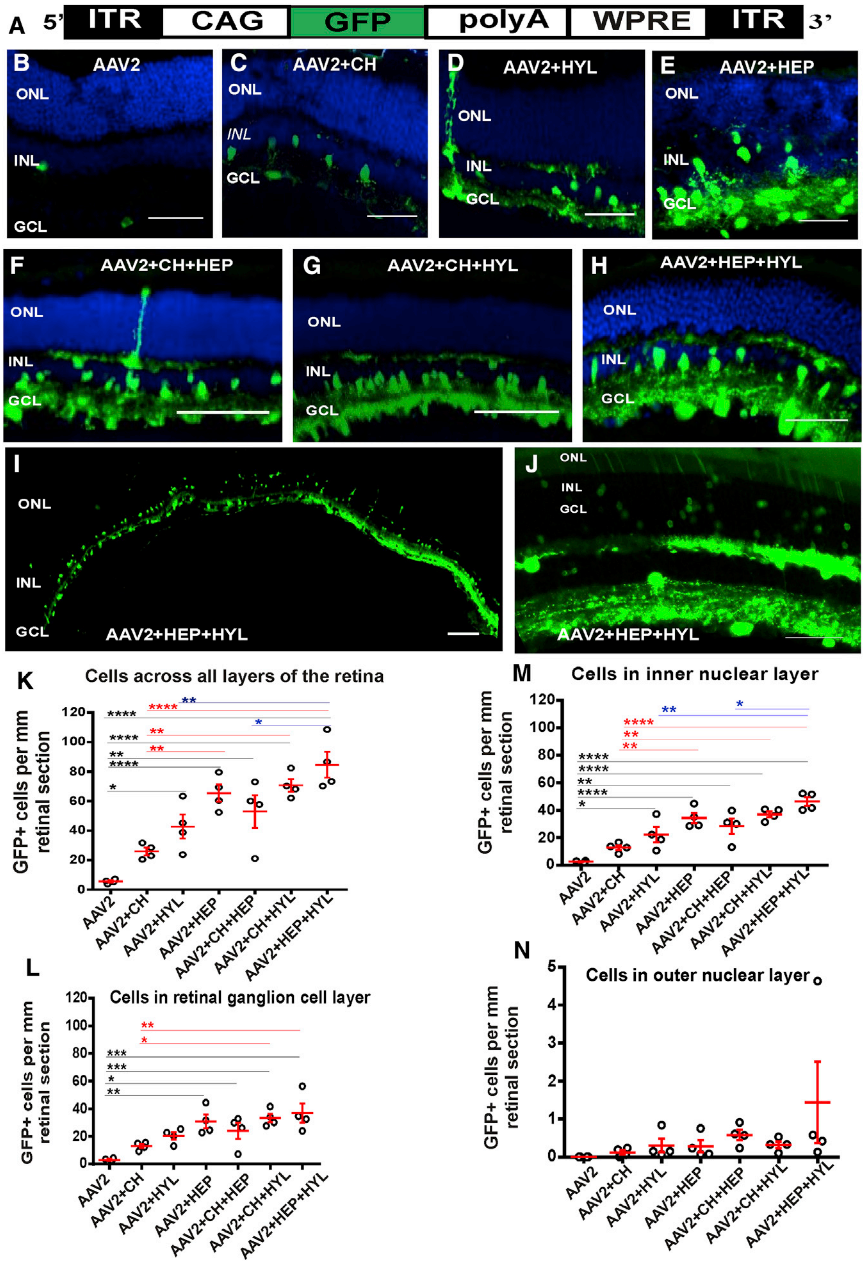

Figure 1. Transduction Efficiency in Wild-Type Retinas of Low-Dose AAV2-CAG-GFP Vector in Combination with Glycosidic Enzymes

(A) Schematic of the AAV2-CAG-GFP vector. An EGFP sequence is driven by a hybrid CMV enhancer/chicken β-actin (CAG) promoter. The sequences are flanked by inverted terminal repeats (ITRs) and stabilized by a polyadenylation signal sequence (polyA) and a woodchuck hepatitis posttranscriptional regulatory element (WPRE). (B–H) Exemplar images of transverse sections through a wild-type mouse retina more than 6 weeks after intravitreal delivery of AAV2-CAG-GFP (2×10^8 gc/eye) without glycosidic enzymes (B) or in conjunction with glycosidic enzymes: chondroitin ABC lyase (CH, C), hyaluronan lyase (HYL, D), and heparinase III (HEP, E) and their combinations (CH+HEP, F), (CH+HYL, G), and (HEP+HYL, H). Nuclei were counterstained with DAPI (blue). (I and J) Exemplar images of sections through a wild-type mouse retina more than 6 weeks after intravitreal delivery of 2×10^8 gc/eye AAV2-CAG-GFP in conjunction with heparinase III and hyaluronan lyase combined, demonstrating the extent of transgene expression in a cryosection across the entire retina (low-magnification image, 4× objective; I) and part of a retina where GFP expression is present in the outer nuclear layer ONL (high-magnification image, 20× objective; J). Scale bars, 50 μm. (K–N) Quantitative analyses of the transduction efficiency of the vector in (A), showing GFP+ cell counts per millimeter of retinal section: across all retinal layers (K), in the GCL (L), in the INL (M), and in the ONL (N). Ordinary one-way ANOVA with Turkey's multiple comparison's test was used to compare counts between groups (*$p < 0.05$, **$p < 0.01$, ***$p < 0.001$, ****$p < 0.0001$).

efficient retinal transduction.[11] Enzymatic digestion of the ILM with the non-specific protease Pronase E also enhanced retinal transduction, suggesting that the ILM forms an important barrier to vector penetration.[12]

Here we describe an optimized approach to increasing retinal transduction of intravitreally delivered unaltered AAV2 in mice achieved by co-injecting glycosidic enzymes. We performed quantitative and qualitative analyses of the transduction efficiency of AAV2 (carrying a reporter gene, GFP) in conjunction with several glycosidic enzymes, including chondroitin ABC lyase, hyaluronan lyase, heparinase III, and combinations thereof, and found that a combination of heparinase III and hyaluronan lyase produced the greatest improvement in retinal penetration by the AAV2 vector (including modest expression in photoreceptors) and overall the highest level of transduction in intact wild-type retina. Robust transgene expression was also observed using these enzymes with intravitreal AAV2 in the degenerate retina of *rd1* mice, a model of advanced retinal degeneration, after both untargeted delivery and when GFP was selectively targeted to ON-bipolar cells. Safety studies demonstrated that retinal function was unaffected in the short, intermediate, and long-term phases after enzyme treatment.

RESULTS

Glycosidic Enzymes Increase the *In Vivo* Transduction Efficiency of AAV2 from the Vitreous

To characterize expression of the reporter gene (GFP) mediated via a low-dose of the AAV2 vector, we injected 2×10^8 genomic counts (gc)/eye of AAV2-CAG-GFP vector (comprising an EGFP coding sequence under the control of a ubiquitous promotor, Figure 1A)

into the vitreous of adult wild-type mice alone or in combination with glycosidic enzymes. As expected, with AAV2-CAG-GFP alone, there was weak gene expression, and this was restricted to the inner retina (Figure 1B; Figure S1A).[11,12,25] By contrast, when AAV2-CAG-GFP was injected in conjunction with the glycosidic enzymes chondroitin ABC lyase (Figure 1C), hyaluronan lyase (Figure 1D; Figure S1B), or heparinase III (Figure 1E; Figure S1C), there was a marked increase in GFP expression in the retinal ganglion cell layer (GCL) and inner nuclear layer (INL), confirming our previous findings.[11] Next we tested various combinations of enzymes, including chondroitin ABC lyase+heparinase III (Figure 1F), chondroitin ABC lyase+hyaluronan lyase (Figure 1G), and heparinase III+hyaluronan lyase) (Figure 1H) and found that they further enhanced GFP expression. The strongest transduction was achieved with a combination of heparinase III and hyaluronan lyase (Figure 1H), which produced robust GFP expression throughout the GCL and INL (Figure 1I), with some regions also having modest outer retinal transduction (Figure 1J). Quantitative assessment of the transduction efficiency of AAV2-CAG-GFP showed a significant increase in the number of GFP+ cell bodies per millimeter of retinal section with addition of the glycosidic enzymes hyaluronan lyase or heparinase III (ordinary one-way ANOVA with Turkey's multiple comparisons test for comparing counts between groups; HYL, $p < 0.05$; heparinase III, $p < 0.0001$) but not with chondroitin ABC lyase ($p > 0.05$) compared with AAV2 alone (Figure 1K). Among these, heparinase III produced significantly more retinal transduction than chondroitin ABC lyase alone (ordinary one-way ANOVA with Turkey's multiple comparisons test, $p < 0.01$).

When combinations of glycosidic enzymes were used, they also demonstrated a significant improvement in transduction efficiency (chondroitin ABC lyase+heparinase III, $p < 0.01$; chondroitin ABC lyase+hyaluronan lyase, $p < 0.0001$; heparinase III+hyaluronan lyase, $p < 0.0001$) compared with eyes not treated with enzymes. In particular, heparinase III and hyaluronan lyase resulted in the highest counts of GFP+ cells, with a ~17-fold increase compared with unenhanced AAV2-CAG-GFP-mediated transduction ($p < 0.0001$). This increase was significant in both GCL (Figure 1L; $p < 0.001$) and INL (Figure 1M; $p < 0.0001$) cells but not in outer nuclear layer (ONL) cell bodies (Figure 1N).

High-Dose AAV2 Vector in Conjunction with the Heparinase III and Hyaluronan Lyase Enzymes in Wild-Type Mice

To determine the extent of transduction achievable with this technique, we next applied the most effective combination (heparinase III + hyaluronan lyase) with a higher dose of vector. For these experiments, we injected wild-type mice with the combination of heparinase III and hyaluronan lyase, along with a higher dose of AAV2-CAG-GFP (3×10^{10} gc/eye). No green fluorescent signal was observed in retinas from eyes injected with PBS alone (Figure 2A). Increasing the vector dose in the presence of the glycosidic enzymes resulted in strong pan-retinal transduction of cells in the GCL and INL (Figures 2C–2E) compared with injection of a high-dose vector alone (Figure 2B). In addition, outer retinal transduction (ONL) was observed, albeit at a lower level (Figure 2C, bottom image), with longer stretches of modest GFP expression (Figure 2F) and some areas of relatively high expression (Figure 2G). This patchy expression is potentially due to non-homogeneous diffusion of the enzymes and vector through the vitreous, leading to more efficient retinal transduction near the injection site.

AAV2 Vector in Conjunction with Glycosidic Enzymes Leads to Robust Expression of the Reporter Gene in *rd1* Retinas

We next asked whether the enzyme approach was also suitable for the degenerated retina. For these experiments, we investigated AAV2 transduction in *rd1* retinas—a model of advanced retinal degeneration. We injected AAV2-CAG-GFP vector (3×10^{10} gc/eye) with heparinase III and hyaluronan lyase into the vitreous of adult *rd1* mice (> 8 weeks of age). When retinas were harvested ~6 weeks after injection, robust reporter gene expression was observed in cells of the GCL and optic nerve and in the INL (Figures 3A, 3C, and 3D) compared with AAV2-CAG-GFP alone (Figure 3B). The expression in the GCL was uniform, whereas INL transgene expression, although pan-retinal, displayed more varied density and depth (Figure S2A). In further experiments, we injected AAV2 with an ON-bipolar specific promoter driving GFP expression (AAV2-grm6-GFP; Figure 3E) in conjunction with glycosidic enzymes and found, as expected, expression restricted to cells in the INL (Figures 3F–3H; Figure S2B). The grm6/SV40 enhancer promoter sequence has been shown in several studies to drive expression exclusively in ON-bipolar cells;[26–31] these were identified on the basis of their axon terminals ending in the proximal part of the inner plexiform layer or ON sublamina and by co-staining with markers of ON-bipolar cells, including protein kinase Cα (PKCα) and TrpM1L.

Short-, Intermediate-, and Long-Term Effects of Glycosidic Enzymes on Retinal Function

We evaluated the safety of enzyme treatments by examining their short-, medium-, and long-term effects on retinal function in wild-type animals. All histological sections appeared to be morphologically intact (Figures 1, 2, and 3), so the enzymes did not produce an obvious disruption of the retinal architecture. We investigated whether the glycosidic enzymes had any effect upon retinal function, as assessed by electroretinograms (ERGs) elicited by flash stimuli 1 week after injection (Figures 4A–4D). We observed no significant differences in the mean amplitude of the a-wave (assessing photoreceptor function; Figures 4A and 4B) or the b-wave (assessing bipolar cell function; Figures 4C and 4D) under scotopic (Figures 4A and 4C) or photopic (Figures 4B and 4D) conditions in enzyme-treated (hyaluronan lyase, heparinase III, or heparinase III combined with hyaluronan lyase) compared with control (PBS or AAV2-CAG-GFP without enzymes) eyes 1 week after intravitreal injections (ordinary one-way ANOVA with Dunnett's multiple comparisons test comparing differences in the mean between control and treated groups ($p > 0.05$ for each condition; Figures 4A–4D). These findings confirm that retinal functions, specifically photoreceptor and bipolar cell functions, remain unchanged shortly (1 week) after treatment.

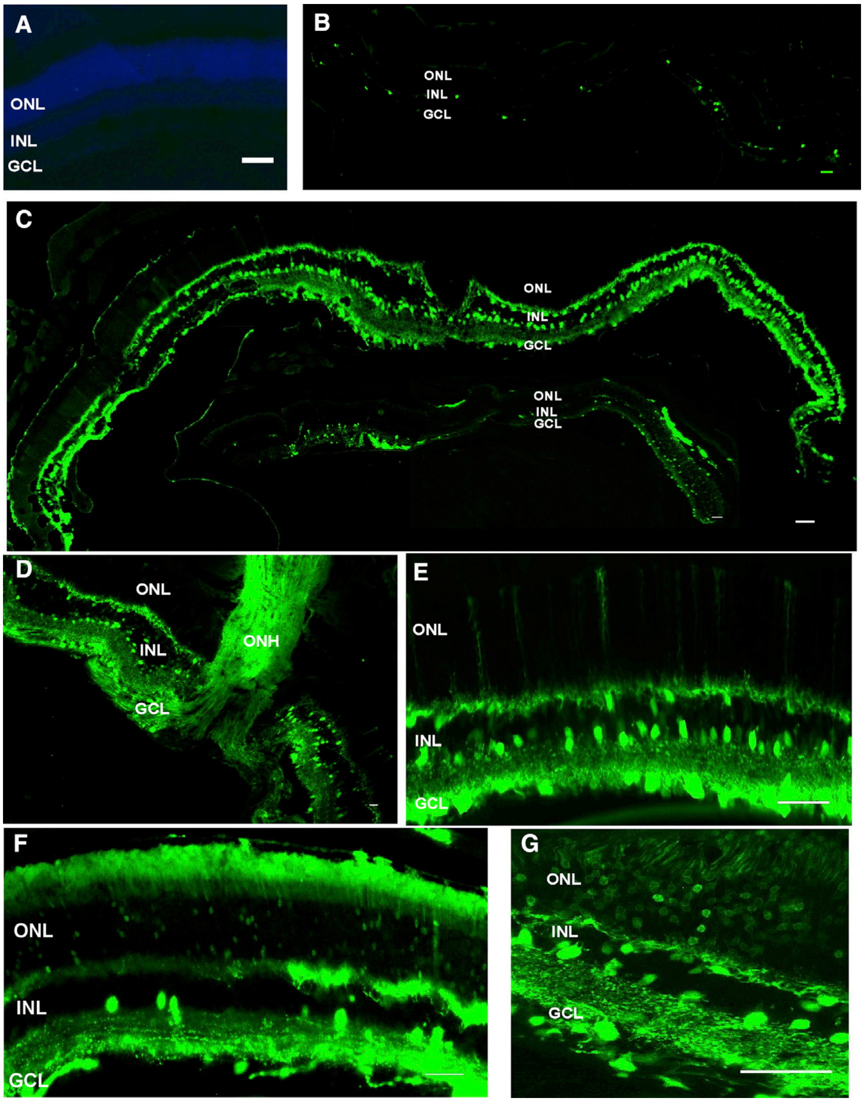

Figure 2. Transduction Efficiency in Wild-Type Retinas of the High-Dose AAV2-CAG-GFP Vector in Combination with Heparinase III and Hyaluronan Lyase

(A–G) Exemplar images of transverse sections through an adult wild-type mouse retina more than 6 weeks after intravitreal delivery of PBS (A), AAV2-CAG-GFP vector (B, 3×10^{10} gc/eye), and AAV2-CAG-GFP vector in conjunction with HEP and HYL (C-G). Two cryosections are shown in (C), depicting the extent of unamplified GFP expression (green) along the ganglion cell layer (GCL) and inner nuclear layer (INL, top image) and a more patchy expression in the outer nuclear layer (ONL, bottom image). Robust GFP expression is observed in the GCL and INL (D and E), with some areas of particularly high expression in Müller cells (E), whereas more variable expression is found in the ONL (F), with some patches of strong expression (G). Nuclei were counterstained with DAPI (blue, A). Scale bars, 50 μm.

Next we assessed retinal function 6 weeks after treatment. As with week 1, we found no significant differences in the mean amplitude of scotopic a-wave (Figure 4E), photopic a-wave (Figure 4F), scotopic b-wave (Figure 4G), or photopic b-wave (Figure 4G) amplitudes in enzyme-treated compared with control (PBS- or AAV2-CAG-GFP-treated without enzymes) eyes (ordinary one-way ANOVA with Dunnett's multiple comparisons test for comparing differences in the mean between control and treated groups; $p > 0.05$ for each condition at 6 weeks; Figures 4E–4H; paired t test $p > 0.05$ also for comparison of week 1 and week 6 data for each group). Notably, a few eyes (11 of 82, marked with stars across enzyme-treated and control groups; Figures 4E–4H) with lower a- and b-wave amplitudes had persistent intravitreal hemorrhage at 1 week that was caused by the injection procedure. In the majority of cases (8 of 11), this completely resolved by week 6 and was associated with a recovery in ERG amplitudes.

We examined retinal function in terms of photosensitivity and recorded scotopic ERGs not only with a bright flash (13.85 log photons/cm^2/s) but also at lower retinal irradiances spanning 9 log units (from neutral density [ND] 0–ND8) for enzyme-treated (heparinase III+hyaluronan lyase) and control (PBS) eyes. We found no differences in irradiance-response curves (IRCs) between enzyme-treated and PBS-injected eyes at week 1 or 6 after treatment (Figure 4I; $p > 0.05$, ordinary one-way ANOVA with Turkey's multiple comparisons test for comparing differences in the mean between groups at each irradiance).

Because ganglion cell activity is not directly assessed by the ERG, we recorded the pupillary light reflex (PLR) at a range of retinal irradiances (spanning 6 log units) in wild-type animals in which one eye was treated with enzymes and the other with PBS (internal control) 6 weeks after intravitreal injection. No difference in PLR function was observed at any irradiance in enzyme-treated compared with control eyes ($p > 0.05$, paired t test between enzyme- and PBS-treated eyes at each irradiance), demonstrating intact retinal ganglion cell function following treatment with glycosidic enzymes.

Last, we examined the long-term effects of combined heparinase III and hyaluronan lyase on retinal function by repeating our safety assessments after intravitreal injections in wild-type mice. ERGs at 12 months post-treatment (using a separate cohort of mice; Figures 5A–5E) showed that, again, there were no significant differences in the mean amplitude of scotopic and photopic a-waves (Figures 5A and 5B) or scotopic and photopic b-waves (Figures 5C and 5D) between enzyme-treated and control eyes 12 months after injections

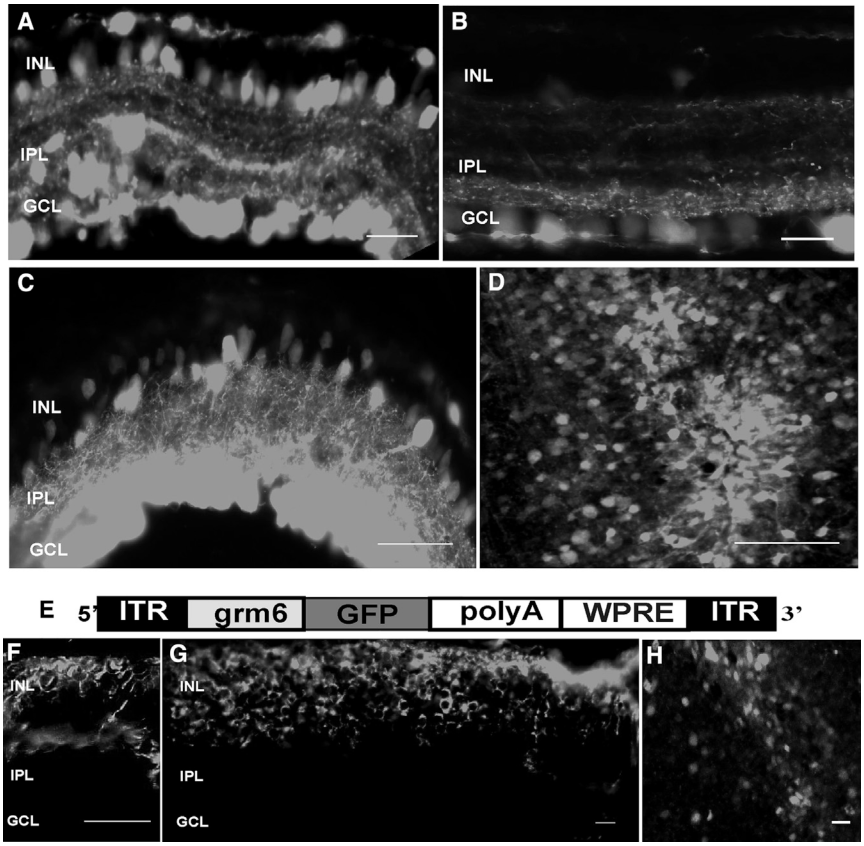

Figure 3. Transduction Efficiency Profile in *rd1* Retinas of High-Dose AAV2-CAG-GFP and AAV2-grm6-CAG Vectors in Combination with Heparinase III and Hyaluronan Lyase

(A–D) Exemplar images of transverse sections through an *rd1* mouse retina more than 6 weeks after intravitreal delivery of AAV2-CAG-GFP vector (3×10^{10} gc/eye) in conjunction with HEP and HYL (A, ~8-μm-thick section, as are the other sections in the image, apart from C, which is ~50 μm in thickness) or AAV2 alone (B). Strong GFP expression (gray) is observed in the cells of the GCL and INL. (D) Exemplar image of an area from a retinal whole mount (oriented with the GCL facing upward) showing robust GFP expression. (E–H) Schematic of the AAV2-grm6-GFP vector with the ON-bipolar cell-specific promoter grm6 (E), which was delivered intravitreally to *rd1* mice at 3×10^{10} gc/eye. (F and G) Exemplar images of transverse sections (F, ~8 μm in thickness, and G, ~30 μm in thickness) through an *rd1* mouse retina more than 6 weeks after intravitreal delivery of the AAV2-grm6-GFP vector in conjunction with HEP and HYL. Robust GFP expression (gray) is observed in the cells of the INL. (H) Exemplar image of an area from a retinal whole mount (oriented with the INL facing upward), showing strong GFP expression. Scale bars, 50 μm.

(Figures 5A–5D; $p > 0.05$; ordinary one-way ANOVA with Turkey's multiple comparisons test for comparing differences in the mean between groups at 6 weeks and 12 months). In addition, we compared the IRC for scotopic ERGs between enzyme- and PBS-treated eyes 12 months after injection and found no significant differences in maximum b-wave amplitude at a range of irradiances tested (Figure 5E; $p > 0.05$; ordinary one-way ANOVA with Turkey's multiple comparisons test for comparing differences in the mean between groups at 6 weeks and 12 months).

To assess any detrimental long-term effects of enzymes on retinal ganglion cell (RGC) function, we re-recorded the PLR 6 months after treatment. We observed no significant shift in the irradiance response curves between enzyme- and PBS-treated eyes at a range of irradiances tested (Figure 5F; $p > 0.05$; paired t test for comparing mean pupillary constriction between enzyme- and PBS-treated eyes at each irradiance at 6 months).

DISCUSSION

We have demonstrated that intravitreal injection of glycosidic enzymes is an effective method of increasing AAV2-mediated transduction of the retina. The single enzyme that produced the largest effect was heparinase III, which, in combination with heparinase III and hyaluronan lyase, produced the largest effect in wild-type and degenerate retinas, as assessed by fluorescence derived from a GFP reporter gene. We observed a diverse population of transduced cells and penetration of deeper layers of the retina, including the outer retinal layer; when we used an ON-bipolar cell-specific promoter, we confined this expression to the INL. Short- and long-term safety studies, assessing retinal function *in vivo*, demonstrated that retinas can tolerate this enzymatic treatment and that their function and sensitivity remain unchanged for at least 12 months after the intraocular injection.

The approach described here advances our previous work, where we compared enzyme activity based on relative fluorescence in retinal whole mounts 2 weeks after AAV2-GFP injections. In these experiments, we observed an increase in retinal fluorescence compared with controls (without enzyme) after intravitreal injection of CAG-AAV2-GFP in conjunction with chondroitin ABC lyase or heparinase III and a weak effect from hyaluronan lyase 2 weeks after injections. Here we show that a higher dose of hyaluronan lyase (0.125 compared with 0.05 units per eye used in the earlier study) led to a significant increase in retinal transduction (as determined by the number of GFP+ cells) 6 weeks after injections. The increase was even greater than that produced by chondroitin ABC lyase alone, although a combination of two enzymes led to an additive increase in the overall transduction rate.

To reach photoreceptors from the vitreous, the AAV needs to diffuse through the vitreous away from the site of injection, pass through the ILM, and move through the retinal matrix into the outer retina. Glycosaminoglycans are long, highly charged polysaccharide chains

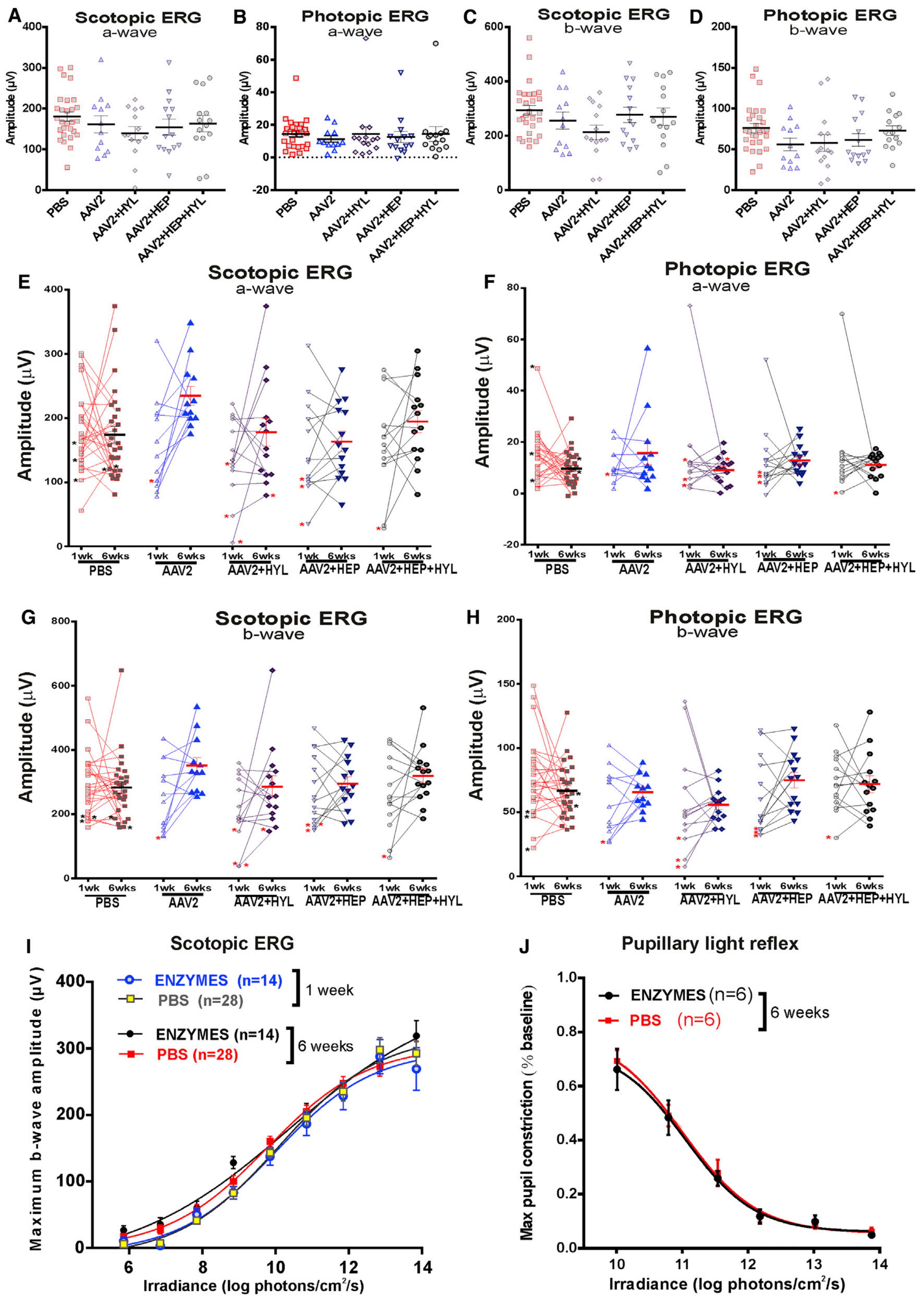

(legend on next page)

found in the ECM and on cell surfaces. With the exception of hyaluronan, they are all synthesized and covalently linked onto core proteins (forming proteoglycans). Glycosaminoglycan chains, including hyaluronan, heparan sulfate, chondroitin sulfate, and dermatan sulfate, are present throughout the retina.[32] Hyaluronan lyase, by its specific ability to cleave hyaluronan, facilitates the movement of the AAV into and through the retina.[32] The highest concentration of hyaluronan is in the vitreous, degrading the vitreous hyaluronan may facilitate diffusion of the AAV through the vitreous cavity, allowing widespread delivery to the ILM at the vitreo-retinal junction. Heparinase III, by its action on heparan sulfate chains of heparan sulfate proteoglycans (HSPGs), abundant in the ILM as well as in other retinal layers (including the nerve fiber layer, the inner and outer plexiform layers, and the interphotoreceptor matrix),[32,33] could make these retinal layers more porous and thus improve the *trans*-retinal penetration of the viral vector. In addition, AAV2 can bind heparan sulfate, so heparan sulfate in extracellular structures such as the ILM could sequester AAV2 and prevent its movement through the retina. However, although heparan sulfate is thought to be a cell surface receptor for AAV2, digestion with heparinase III did not prevent entry of the AAV into retinal cells. This increased tissue penetration may also enable the use of relatively low vector doses (e.g., 3×10^{10} gc/eye), reducing the chance of an adverse immune response.[34] Interestingly, the 7m8 variant of AAV2, which produces outer nuclear transduction following intravitreal injection, has a lower affinity for binding to its primary cell surface receptor, heparan sulfate, and may therefore also have a lower affinity for heparan sulfate in the ILM.[35,36] In addition, intravitreal delivery of non-AAV2-based vectors, which do not have a strict requirement for interaction with the heparan sulfate receptor, might lead to even better pan-retinal transduction when delivered in conjunction with glycosidic enzymes. To this extent, intravitreal delivery of AAV5 in conjunction with enzymatic digestion of ILM with pronase leads to very good transduction of the mouse retina.[12]

The safety profiles of the glycosidic enzymes used here are very encouraging. Electroretinography (ERG) confirmed unaltered a- and b-waves (which specifically tested photoreceptor and bipolar cell function, respectively) short-, intermediate-, and long-term after the treatment, whereas pupillometry confirmed that retinal ganglion cell activity was retained. Retinal photosensitivity remained unchanged, as determined by electroretinograms and pupillometry at a range of irradiances tested. Glycosidic enzymes (hyaluronidases and a chondroitinase) have previously been tested in animal models for pharmacological vitreolysis without reported adverse effects,[37–39] and a highly purified ovine hyaluronidase, Vitrase (ISTA Pharmaceuticals, California, USA) has been used in a clinical trial aiming to aid the dispersion of vitreous hemorrhage.[40] On a molecular level, using electron microscopy of monkey ILM digests, chondroitin ABC lyase was shown to have no effect on the morphology of the retina, whereas testicular hyaluronidase (which has non-specific protease activity) had a mild effect on the ILM, causing the fibrillar meshwork to assume a more irregular pattern.[41]

In this study, we have developed an AAV-mediated treatment with improved retinal transduction by intravitreal injection with an excellent long-term safety profile in rodents. We have recently used this approach to deliver rod opsin to RGC and ON-bipolar cells and successfully rescued vision in an advanced model of retinal degeneration.[42] In addition, it has the potential to effectively deliver AAVs to the outer retina following intravitreal injection, and perhaps further improvements could be made by combining engineered vectors with these glycosidic enzymes.

Nonetheless, compelling evidence exists for barriers to effective pan-retinal transduction of non-human primate retina by intravitreal injection. In comparison with the rodent retina, these barriers involve even greater challenges and include dilutional effects of mixing vector solution with vitreous; the risk of vector neutralization and inflammatory responses in non-immune-privileged vitreous humor;[43–45] and the physical barrier of the highly impenetrable primate ILM, where only a small foveal ring becomes transduced following intravitreal injection of AAV vectors.[16,23,45] Novel strategies are therefore exploring new ways of manipulating the ILM either surgically, enzymatically (as proposed in this study), or by injecting the vector under the ILM.[46] It is possible that such approaches will lead to even greater immune responses because they could result in the unimpeded passage of immune cells between the retina and vitreous. Furthermore, rodent

Figure 4. Short- and Intermediate-Term Effects of Glycosidic Enzymes on Retinal Function in Wild-Type Animals

(A–H) Electroretinograms following intravitreal injections of PBS, AAV2-CAG-GFP (AAV) alone, and AAV2-CAG-GFP (3×10^{10} gc/eye) in combination with HEP+HYL. Dark-adapted (scotopic, A and C) and light-adapted (photopic, B and D) electroretinogram (ERG) recordings show the distribution of maximum a-wave (mean ± SEM, A and B) and b-wave (mean ± SEM, C and D) amplitude 1 week after intravitreal injection. Following injection of the glycosidic enzymes, there was no significant change in a-wave or b-wave amplitude compared with vector only- or PBS-injected eyes ($p > 0.05$, ordinary one-way ANOVA with Dunnett's multiple comparisons test). (E–H) Dark-adapted (scotopic, E and G) and light-adapted (photopic, F and H) ERG recordings showing paired comparison of maximum a-wave (E and F) and b-wave (G and H) amplitude 1 and 6 weeks after intravitreal injection. Stars signify the presence of an intravitreal hemorrhage. Horizontal bars/error bars, mean ± SEM. Glycosidic enzyme injections did not significantly change a-wave or b-wave amplitude compared with vector alone- or PBS-injected eyes 6 weeks after treatment ($p > 0.05$, ordinary one-way ANOVA with Turkey's multiple comparisons test for comparing means between all groups at 1 and 6 weeks). (I and J) Retinal photosensitivity after intravitreal delivery of hyaluronan lyase and heparinase III (ENZYMES) or PBS alone, as measured by the irradiance-response curve for maximum b-wave amplitude in the scotopic ERG (I, 1 and 6 weeks after treatment) or maximum pupillary constriction in the pupillary light reflex (PLR) (J, 6 weeks after treatment) at a range of retinal irradiances. There is no significant difference in scotopic ERGs (I, 1 or 6 weeks after treatment) or in the PLR (J, 6 weeks after treatment) in eyes that were treated with enzymes and those that were not. The data for the PLR are normalized to pupil size immediately preceding light onset (10 s white light). Values are mean ± SEM, with n indicating the number of mice examined. The data are fitted with a sigmoidal function (I, $p > 0.05$, ordinary one-way ANOVA with Turkey's multiple comparisons test for comparing differences in the mean between groups at each irradiance for the scotopic ERG; J, $p > 0.05$, paired t test between eyes that were and were not treated with the enzymes at each irradiance for the PLR).

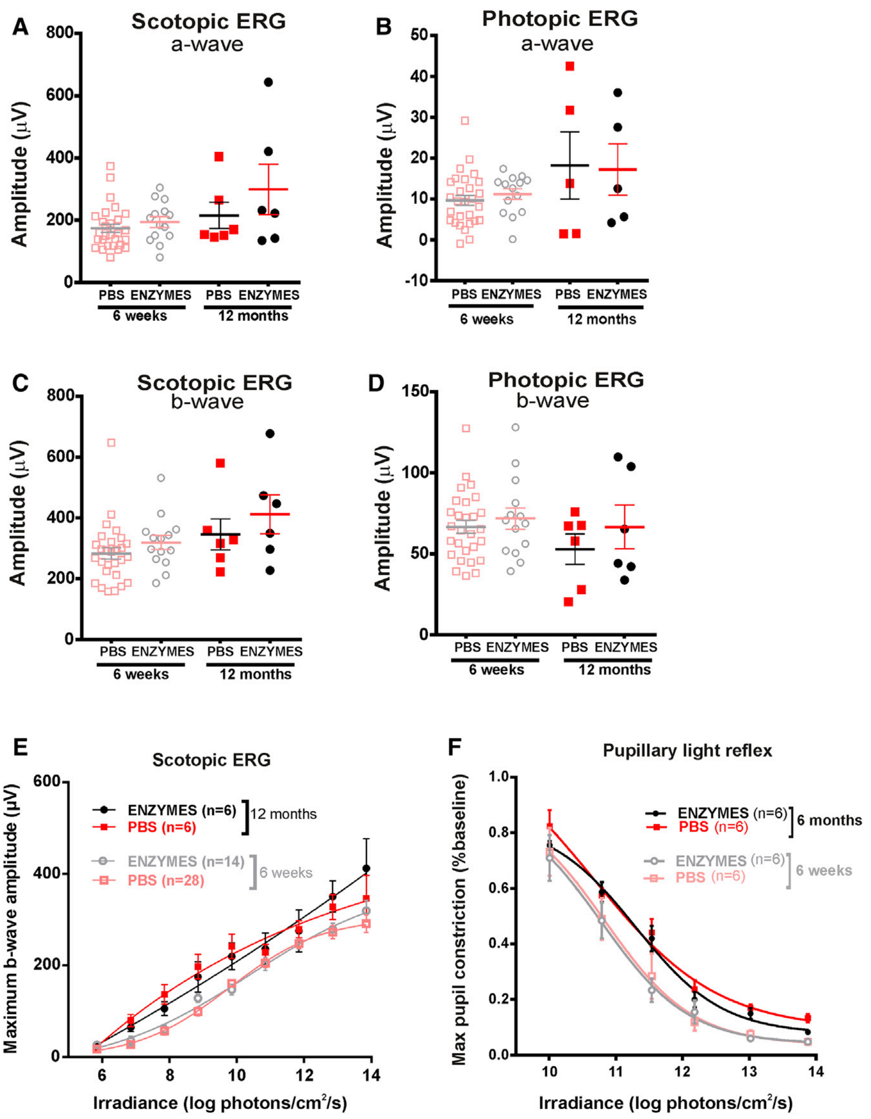

Figure 5. Long-Term Effects of Glycosidic Enzymes on Retinal Function in Wild-Type Animals

(A–D) Dark-adapted (scotopic, A and C) and light-adapted (photopic, B and D) ERG recordings showing the distribution of maximum a-wave (A and B) and b-wave (C and D) amplitude (mean ± SEM) after intravitreal delivery of heparinase III and hyaluronan lyase (ENZYMES) or PBS alone 12 months after treatment. Data obtained 6 weeks after treatment are shown for comparison. Glycosidic enzyme injection showed no significant change in a-wave or b-wave amplitude compared with control (PBS) eyes 12 months after treatment. Two-tailed paired t tests were used to compare the response between enzyme- and PBS-treated eyes at each time point; one-way ANOVA with Turkey's multiple comparison test was used for comparison between all groups. (E and F) Retinal photosensitivity after intravitreal delivery of heparinase III and hyaluronan lyase (ENZYMES) or PBS alone, as measured by irradiance-response curve for maximum b-wave amplitude in the scotopic ERG (E, 12 months after treatment) or maximum pupillary constriction in the PLR (F, 6 months after treatment) at a range of retinal irradiances. Data obtained 6 weeks after treatment are shown for comparison. There is no significant change in the scotopic ERG (E) or in the PLR (F) between enzyme- or PBS only-treated eyes 12 months after treatment. A small decrease in sensitivity is observed for both curves (PBS and ENZYMES) at 6 months compared with 6 weeks (significant at high irradiances; for enzyme curves at 13.8 ($p = 0.02$), 13.0 ($p = 0.01$), and 11.5 ($p = 0.04$) and for PBS curves at 13.8 ($p = 0.007$) and 13.0 ($p = 0.01$) log photons/cm^2/s. The data for the PLR are normalized to pupil size immediately preceding light onset (10 s white light). Values are mean ± SEM, with n indicating the number of mice examined. The data are fitted with a sigmoidal function. Two-tailed paired t test was used to compare responses between enzyme- and PBS-injected eyes at each irradiance. Data obtained at 6 weeks are replotted from Figure 4 for direct comparison.

experiments could underestimate potential adverse immune responses in primate eyes when enzymatic digestion of ILM is combined with intravitreal AAV delivery. However, it is of note that, in a recent study involving surgical manipulation of the ILM in non-human primate retinas followed by intravitreal injection of AAV, the induced inflammatory responses were managed effectively with intravitreal steroids.[47] Ocular inflammation is therefore likely to be one of the drawbacks of intravitreal AAV gene delivery and is likely to require co-administration of high-dose local and/or systemic immunosuppressants to keep it under control.

MATERIALS AND METHODS

Animals

Adult C57BL/6J (wild-type) and C3H/HeJ (*rd1*) mice were used in this study. All animal experiments were conducted in accordance with United Kingdom Home Office regulations for the care and use of laboratory animals, the United Kingdom Animals (Scientific Procedures) Act (1986), and the Animal Welfare Body of the University of Manchester. Animals were kept under a 12-hr light:dark cycle and supplied with food and water *ad libitum.*

Gene Delivery via AAV

The AAV2 vectors used in this study were obtained from Vector Laboratories (Philadelphia, USA). The vectors contained the EGFP gene under the control of a strong ubiquitous pan-neuronal promoter (CAG, a fusion of cytomegalovirus [CMV] early enhancer and chicken β-actin promoter, called AAV2-CAG-GFP; Figure 1A) or a cell-specific ON-bipolar cell promoter (grm6[27]), a fusion of a 200-bp enhancer sequence of the mouse grm6 gene encoding for the ON-bipolar cell-specific metabotropic glutamate receptor,

mGluR6, and an SV40 eukaryotic promoter, called AAV2-grm6-GFP (Figure 3F), flanked by inverted terminal repeat (ITR) domains and stabilized by a polyadenylation signal sequence (poly(A)) and a woodchuck hepatitis posttranscriptional regulatory element (WPRE). Vectors were injected intravitreally in isoflurane-anesthetized mice more than 8 weeks of age.

Prior to injections, pupils were dilated with tropicamide (1%, Chauvin Pharmaceuticals, UK) and phenylephrine (2.5%, Chauvin Pharmaceuticals, UK). A custom-made ultra-fine needle (Hamilton RN needle, 34G, supplied by ESSLAB) was attached to a 5-μL Hamilton glass syringe and passed at 45° through the *pars plana* into the vitreous cavity without retinal perforation. The injection was performed under direct visualization of the needle tip through coverslipped eyes under an operating microscope (Microscopes, USA), carefully avoiding lenticular contact and blood vessels. AAV2-CAG-GFP was injected at a low or high dose and AAV2-grm6-GFP at a high dose only. Eyes that were injected with low-dose vector received 1 μL of 2×10^{11} gc/mL (i.e., 2×10^{8} gc/eye), and those that were injected with high-dose vector received 3 μL of 1×10^{13} gc/mL (i.e., 3×10^{10} gc/eye) diluted in PBS. Each eye that was injected with enzymes received 0.5 μL of PBS containing 0.125 (Sigma) units of chondroitin ABC lyase from *Proteus vulgaris* (E.C. 4.2.2.4), heparinase III from *Flavobacterium heparinum* (E.C. 4.2.2.8), or hyaluronan lyase from *Streptomyces hyalurolyticus* (E.C. 4.2.2.1) singly or in different combinations (all from Sigma-Aldrich, Dorset, UK). Chondroitin ABC lyase digests chondroitin sulfate, dermatan sulfate, and hyaluronan to some extent; heparinase III specifically digests heparan sulfate (and heparin); and hyaluronan lyase specifically digests hyaluronan. The enzyme solutions were freshly made on the day of injection by dissolving the enzymes in sterile PBS. The vector and enzymes were mixed in a syringe immediately before an eye injection and were given in a single combined injection.

Histology

Retrieved eyecups (>6 weeks after injections) were fixed in 4% paraformaldehyde (PFA) in PBS for 24 hr at 4°C after the cornea and lens had been removed anteriorly under a light microscope. The tissue was then washed in PBS prior to incubation in PBS containing 30% sucrose overnight at 4°C. For whole mounts, fixed eyes were washed in PBS, and whole retinas were carefully dissected under a light microscope. Retinas were then flat-mounted with fluorescent mounting medium containing DAPI (Vectashield, Vector Laboratories, Peterborough, UK) to stain cell nuclei. For cryosections, fixed eyes were cryo-protected in optimal cutting temperature medium (Raymond A. Lamb, Eastbourne, UK) and frozen at −80°C until further processing. The cryo-protected retina was sectioned (generally 8–10 μm thickness) on a cryostat (Leica Microsystems) horizontally through the eyecup from ventral to dorsal sides so that each section contained a complete nasal to temporal cross-section of the retina. Slides were stored at −80°C. Prior to analysis, the slides were removed from the freezer, allowed to air-dry at room temperature for 1 hr, and mounted with fluorescent mounting medium containing DAPI (Vectashield, Vector Laboratories, Peterborough, UK) to stain cell nuclei.

Bioimaging

Imaging was performed under an upright fluorescence microscope (Olympus BX51) using several objectives (4×, 10×, or 20×/0.30 Plan Fln), and images were captured using a Coolsnap ES camera (Photometrics) and processed through MetaVue software (Molecular Devices). Images were then analyzed using ImageJ software (https://rsb.info.nih.gov/ij).

Quantitative Analysis of Vector Transduction

For quantification of GFP+ cells in retinal sections, one slide per eye (6–8 sections) for each treatment group (n = 4) was taken for analysis, and all retinal sections were examined on these selected slides. Sections were photographed at 4× using the fluorescence microscope, and the length of each section was measured along the mid-retinal surface in ImageJ. All sections were then re-photographed at 10× to count GFP+ cells. Transduced GFP+ cells were identified on the basis of their laminar location and morphology. GFP+ cells were counted and documented according to the retinal layer in which they were found, including the GCL, INL, and ONL. The quantification was performed with the examiner being blind to the treatment group. The total number of GFP+ cells per eye was divided by the total retinal length for that eye. Data for each group are presented in scatterplots with mean cell count per millimeter retinal section ± SEM. Differences between groups were evaluated using ordinary one-way ANOVA followed by Turkey's multiple comparison's test in GraphPad Prism (V6, GraphPad, USA). Significance was set at $p < 0.05$.

ERG

Retinal function was evaluated in wild-type mice 1 week, 6 weeks, and 12 months after intravitreal injections for all conditions as detailed above. Mice were dark-adapted overnight (>12 hr) and prepared for ERG recordings under dim red light ($<0.6\ \log_{10}\ cd/m^2 > 650$ nm). Anesthesia was induced with an intraperitoneal injection of a mixture of ketamine (75 mg/mL, 10%) and xylazine (13.6 mg/mL, 20%). Pupils were dilated with topical mydriatics (tropicamide 1% and phenylephrine 2.5%; Chauvin Pharmaceuticals, UK) prior to placement of a corneal contact lens-type electrode held in place by a drop of hydroxypropyl methylcellulose solution (0.5%, Alcon Laboratories, UK). The mice were placed onto a silver wire bite bar that provided head support and acted as a ground. A needle reference electrode (Ambu, Neuroline) was inserted subcutaneously into the left cheek. Electrodes were connected to a Windows personal computer (PC) via a signal conditioner (model 1902 Mark III, CED, UK), which differentially amplified (×3,000) and filtered (band-pass filter cutoff, 0.5 to 200 Hz) the signal, and a digitizer (model 1401, CED). ERG signals were averaged three to six times to reduce noise. Core body temperature was maintained throughout the procedure with a homeothermic heat mat (Harvard Apparatus).

Light Protocol

Both dark-adapted (scotopic) and light-adapted (photopic) ERGs were recorded. Scotopic ERGs were performed in the dark and elicited by 15-ms full-field flashes produced by a light source (cold white light-emitting diode [LED], 800 mW, Thorlabs) fitted with ND filters

in an ascending intensity series (retinal irradiances in the range of 13.85–5.85 log photons/cm^2/s). Average response waveforms for each individual were produced from between 30 and 6 stimulus repeats applied at inter-stimulus intervals ranging from 1,500 ms at the dimmest intensities to 30 s at the brightest intensities to avoid significant bleaching of the visual pigment. Photopic ERGs were performed under room illumination and elicited by bright white flashes (10-μs duration at ND0; peak retinal irradiance, 13.85 log photons/cm^2/s) recorded over 20 min at a frequency of 0.75 Hz, presented against a rod-saturating background white light. The amplitude of the a-wave was measured from the baseline prior to stimulus onset to the primary trough of negative polarity voltage. The amplitude of the b-wave was determined from the a-wave trough to the maximum of the secondary positive peak. Data for each group are presented in scatterplots with mean amplitude ± SEM. IRCs were fitted with sigmoidal function. Statistical differences between groups were evaluated in GraphPad Prism (V6, GraphPad, USA) using ordinary one-way ANOVA followed by Dunnett's post-test (comparing group means with the control PBS or AAV2 group), Turkey's post-test (multiple comparisons of group means between all groups), or, in instances when only 2 groups were compared, two-tailed paired t test to compare responses between enzyme- and PBS only-injected eyes. Significance was set at $p < 0.05$.

Pupillometry

PLR was measured in wild-type mice 6 weeks and 6 months after injections, for all conditions, as detailed above. Mice were dark-adapted overnight (> 12 hr) before the recordings. Light stimuli were provided by a 150-W metal halide lamp (Phillips, USA) and transmitted along a fiber-optic bundle to an integrating reflective sphere, which provided uniform light at the mouse cornea (similar to what has been described previously by Enezi et al.[47]). Consensual PLR was recorded in unanesthetized, lightly scruffed mice under infrared conditions with an infra-red sensitive charge-coupled device (CCD) camera fitted with a 10× macro lens and an infra-red filter. An intervening shutter controlled stimulus timing. A single trial lasted 20 s: 5 s light OFF, 10 s light ON, 5 s light OFF. The intensity of the light was controlled by ND filters, and mice were subjected to white light exposure in an ascending intensity series, with individual trials being separated by at least 5 min. Retinal irradiance values ranged from 13.8 (ND0) to 10.01 (ND5) log photons/cm^2/s. Pupillary responses were quantified from the video images using ImageJ software. Data were normalized to pupil area immediately preceding light onset and reported as maximum pupillary constriction, mean ± SEM. IRCs were fitted with sigmoidal function. Statistical differences between groups were evaluated in GraphPad Prism (V6, GraphPad, USA) using a two-tailed paired t test to compare response between enzyme- and PBS only-injected eyes. Significance was set at $p < 0.05$.

SUPPLEMENTAL INFORMATION

Supplemental Information includes two figures and can be found with this article online at https://doi.org/10.1016/j.omtm.2017.12.002.

AUTHOR CONTRIBUTIONS

J.C.-K., P.N.B., and R.J.L. designed the research. J.C.-K. performed intraocular injections, ERGs, pupillometry, retinal histology, data processing, and analysis. R.A.B. assisted with pupillometry. N.M. performed ERG recordings 12 months after treatment with assistance from R.A.B. J.C.-K. wrote and revised the manuscript with comments from all authors. P.N.B. and R.J.L. supervised the research.

ACKNOWLEDGMENTS

We thank Jonathan Wynne for technical assistance. J.C.-K. was supported by Medical Research Council Clinical Research Training Fellowship G1000268/1. We also acknowledge grant support from the ERC (268970 to R.J.L.) and MRC (MR/N012992/1 to R.J.L. and P.N.B.).

REFERENCES

1. Bainbridge, J.W., Smith, A.J., Barker, S.S., Robbie, S., Henderson, R., Balaggan, K., Viswanathan, A., Holder, G.E., Stockman, A., Tyler, N., et al. (2008). Effect of gene therapy on visual function in Leber's congenital amaurosis. N. Engl. J. Med. *358*, 2231–2239.

2. Maguire, A.M., Simonelli, F., Pierce, E.A., Pugh, E.N., Jr., Mingozzi, F., Bennicelli, J., Banfi, S., Marshall, K.A., Testa, F., Surace, E.M., et al. (2008). Safety and efficacy of gene transfer for Leber's congenital amaurosis. N. Engl. J. Med. *358*, 2240–2248.

3. Hauswirth, W.W., Aleman, T.S., Kaushal, S., Cideciyan, A.V., Schwartz, S.B., Wang, L., Conlon, T.J., Boye, S.L., Flotte, T.R., Byrne, B.J., and Jacobson, S.G. (2008). Treatment of leber congenital amaurosis due to RPE65 mutations by ocular subretinal injection of adeno-associated virus gene vector: short-term results of a phase I trial. Hum. Gene Ther. *19*, 979–990.

4. Maguire, A.M., High, K.A., Auricchio, A., Wright, J.F., Pierce, E.A., Testa, F., Mingozzi, F., Bennicelli, J.L., Ying, G.S., Rossi, S., et al. (2009). Age-dependent effects of RPE65 gene therapy for Leber's congenital amaurosis: a phase 1 dose-escalation trial. Lancet *374*, 1597–1605.

5. Bainbridge, J.W., Mehat, M.S., Sundaram, V., Robbie, S.J., Barker, S.E., Ripamonti, C., Georgiadis, A., Mowat, F.M., Beattie, S.G., Gardner, P.J., et al. (2015). Long-term effect of gene therapy on Leber's congenital amaurosis. N. Engl. J. Med. *372*, 1887–1897.

6. Jacobson, S.G., Aleman, T.S., Cideciyan, A.V., Sumaroka, A., Schwartz, S.B., Windsor, E.A., Traboulsi, E.I., Heon, E., Pittler, S.J., Milam, A.H., et al. (2005). Identifying photoreceptors in blind eyes caused by RPE65 mutations: Prerequisite for human gene therapy success. Proc. Natl. Acad. Sci. USA *102*, 6177–6182.

7. Wright, A.F., Chakarova, C.F., Abd El-Aziz, M.M., and Bhattacharya, S.S. (2010). Photoreceptor degeneration: genetic and mechanistic dissection of a complex trait. Nat. Rev. Genet. *11*, 273–284.

8. Jacobson, S.G., Cideciyan, A.V., Ratnakaram, R., Heon, E., Schwartz, S.B., Roman, A.J., Peden, M.C., Aleman, T.S., Boye, S.L., Sumaroka, A., et al. (2012). Gene therapy for leber congenital amaurosis caused by RPE65 mutations: safety and efficacy in 15 children and adults followed up to 3 years. Arch. Ophthalmol. *130*, 9–24.

9. Jacobson, S.G., Acland, G.M., Aguirre, G.D., Aleman, T.S., Schwartz, S.B., Cideciyan, A.V., Zeiss, C.J., Komaromy, A.M., Kaushal, S., Roman, A.J., et al. (2006). Safety of recombinant adeno-associated virus type 2-RPE65 vector delivered by ocular subretinal injection. Mol. Ther. *13*, 1074–1084.

10. Nork, T.M., Murphy, C.J., Kim, C.B., Ver Hoeve, J.N., Rasmussen, C.A., Miller, P.E., Wabers, H.D., Neider, M.W., Dubielzig, R.R., McCulloh, R.J., and Christian, B.J. (2012). Functional and anatomic consequences of subretinal dosing in the cynomolgus macaque. Arch. Ophthalmol. *130*, 65–75.

11. Cehajic-Kapetanovic, J., Le Goff, M.M., Allen, A., Lucas, R.J., and Bishop, P.N. (2011). Glycosidic enzymes enhance retinal transduction following intravitreal delivery of AAV2. Mol. Vis. *17*, 1771–1783.

12. Dalkara, D., Kolstad, K.D., Caporale, N., Visel, M., Klimczak, R.R., Schaffer, D.V., and Flannery, J.G. (2009). Inner limiting membrane barriers to AAV-mediated retinal transduction from the vitreous. Mol. Ther. *17*, 2096–2102.

13. Zhong, L., Li, B., Mah, C.S., Govindasamy, L., Agbandje-McKenna, M., Cooper, M., Herzog, R.W., Zolotukhin, I., Warrington, K.H., Jr., Weigel-Van Aken, K.A., et al. (2008). Next generation of adeno-associated virus 2 vectors: point mutations in tyrosines lead to high-efficiency transduction at lower doses. Proc. Natl. Acad. Sci. USA *105*, 7827–7832.

14. Petrs-Silva, H., Dinculescu, A., Li, Q., Min, S.H., Chiodo, V., Pang, J.J., Zhong, L., Zolotukhin, S., Srivastava, A., Lewin, A.S., and Hauswirth, W.W. (2009). High-efficiency transduction of the mouse retina by tyrosine-mutant AAV serotype vectors. Mol. Ther. *17*, 463–471.

15. Petrs-Silva, H., Dinculescu, A., Li, Q., Deng, W.T., Pang, J.J., Min, S.H., Chiodo, V., Neeley, A.W., Govindasamy, L., Bennett, A., et al. (2011). Novel properties of tyrosine-mutant AAV2 vectors in the mouse retina. Mol. Ther. *19*, 293–301.

16. Yin, L., Greenberg, K., Hunter, J.J., Dalkara, D., Kolstad, K.D., Masella, B.D., Wolfe, R., Visel, M., Stone, D., Libby, R.T., et al. (2011). Intravitreal injection of AAV2 transduces macaque inner retina. Invest. Ophthalmol. Vis. Sci. *52*, 2775–2783.

17. Vandenberghe, L.H., and Auricchio, A. (2012). Novel adeno-associated viral vectors for retinal gene therapy. Gene Ther. *19*, 162–168.

18. Gabriel, N., Hareendran, S., Sen, D., Gadkari, R.A., Sudha, G., Selot, R., Hussain, M., Dhaksnamoorthy, R., Samuel, R., Srinivasan, N., et al. (2013). Bioengineering of AAV2 capsid at specific serine, threonine, or lysine residues improves its transduction efficiency in vitro and in vivo. Hum. Gene Ther. Methods *24*, 80–93.

19. Kay, C.N., Ryals, R.C., Aslanidi, G.V., Min, S.H., Ruan, Q., Sun, J., Dyka, F.M., Kasuga, D., Ayala, A.E., Van Vliet, K., et al. (2013). Targeting photoreceptors via intravitreal delivery using novel, capsid-mutated AAV vectors. PLoS ONE *8*, e62097.

20. Aslanidi, G.V., Rivers, A.E., Ortiz, L., Song, L., Ling, C., Govindasamy, L., Van Vliet, K., Tan, M., Agbandje-McKenna, M., and Srivastava, A. (2013). Optimization of the capsid of recombinant adeno-associated virus 2 (AAV2) vectors: the final threshold? PLoS ONE *8*, e59142.

21. Klimczak, R.R., Koerber, J.T., Dalkara, D., Flannery, J.G., and Schaffer, D.V. (2009). A novel adeno-associated viral variant for efficient and selective intravitreal transduction of rat Müller cells. PLoS ONE *4*, e7467.

22. Bartel, M.A., Weinstein, J.R., and Schaffer, D.V. (2012). Directed evolution of novel adeno-associated viruses for therapeutic gene delivery. Gene Ther. *19*, 694–700.

23. Dalkara, D., Byrne, L.C., Klimczak, R.R., Visel, M., Yin, L., Merigan, W.H., Flannery, J.G., and Schaffer, D.V. (2013). In vivo-directed evolution of a new adeno-associated virus for therapeutic outer retinal gene delivery from the vitreous. Sci. Transl. Med. *5*, 189ra76.

24. Pang, J.J., Dai, X., Boye, S.E., Barone, I., Boye, S.L., Mao, S., Everhart, D., Dinculescu, A., Liu, L., Umino, Y., et al. (2011). Long-term retinal function and structure rescue using capsid mutant AAV8 vector in the rd10 mouse, a model of recessive retinitis pigmentosa. Mol. Ther. *19*, 234–242.

25. Hellström, M., Ruitenberg, M.J., Pollett, M.A., Ehlert, E.M., Twisk, J., Verhaagen, J., and Harvey, A.R. (2009). Cellular tropism and transduction properties of seven adeno-associated viral vector serotypes in adult retina after intravitreal injection. Gene Ther. *16*, 521–532.

26. Masu, M., Iwakabe, H., Tagawa, Y., Miyoshi, T., Yamashita, M., Fukuda, Y., Sasaki, H., Hiroi, K., Nakamura, Y., Shigemoto, R., et al. (1995). Specific deficit of the ON response in visual transmission by targeted disruption of the mGluR6 gene. Cell *80*, 757–765.

27. Kim, D.S., Matsuda, T., and Cepko, C.L. (2008). A core paired-type and POU homeodomain-containing transcription factor program drives retinal bipolar cell gene expression. J. Neurosci. *28*, 7748–7764.

28. Lagali, P.S., Balya, D., Awatramani, G.B., Münch, T.A., Kim, D.S., Busskamp, V., Cepko, C.L., and Roska, B. (2008). Light-activated channels targeted to ON bipolar cells restore visual function in retinal degeneration. Nat. Neurosci. *11*, 667–675.

29. Doroudchi, M.M., Greenberg, K.P., Liu, J., Silka, K.A., Boyden, E.S., Lockridge, J.A., Arman, A.C., Janani, R., Boye, S.E., Boye, S.L., et al. (2011). Virally delivered channelrhodopsin-2 safely and effectively restores visual function in multiple mouse models of blindness. Mol. Ther. *19*, 1220–1229.

30. Cronin, T., Vandenberghe, L.H., Hantz, P., Juttner, J., Reimann, A., Kacsó, A.E., Huckfeldt, R.M., Busskamp, V., Kohler, H., Lagali, P.S., et al. (2014). Efficient transduction and optogenetic stimulation of retinal bipolar cells by a synthetic adeno-associated virus capsid and promoter. EMBO Mol. Med. *6*, 1175–1190.

31. Macé, E., Caplette, R., Marre, O., Sengupta, A., Chaffiol, A., Barbe, P., Desrosiers, M., Bamberg, E., Sahel, J.A., Picaud, S., et al. (2015). Targeting channelrhodopsin-2 to ON-bipolar cells with vitreally administered AAV Restores ON and OFF visual responses in blind mice. Mol. Ther. *23*, 7–16.

32. Clark, S.J., Keenan, T.D., Fielder, H.L., Collinson, L.J., Holley, R.J., Merry, C.L., van Kuppevelt, T.H., Day, A.J., and Bishop, P.N. (2011). Mapping the differential distribution of glycosaminoglycans in the adult human retina, choroid, and sclera. Invest. Ophthalmol. Vis. Sci. *52*, 6511–6521.

33. Keenan, T.D., Clark, S.J., Unwin, R.D., Ridge, L.A., Day, A.J., and Bishop, P.N. (2012). Mapping the differential distribution of proteoglycan core proteins in the adult human retina, choroid, and sclera. Invest. Ophthalmol. Vis. Sci. *53*, 7528–7538.

34. Li, Q., Miller, R., Han, P.Y., Pang, J., Dinculescu, A., Chiodo, V., and Hauswirth, W.W. (2008). Intraocular route of AAV2 vector administration defines humoral immune response and therapeutic potential. Mol. Vis. *14*, 1760–1769.

35. Summerford, C., and Samulski, R.J. (1998). Membrane-associated heparan sulfate proteoglycan is a receptor for adeno-associated virus type 2 virions. J. Virol. *72*, 1438–1445.

36. Kern, A., Schmidt, K., Leder, C., Müller, O.J., Wobus, C.E., Bettinger, K., Von der Lieth, C.W., King, J.A., and Kleinschmidt, J.A. (2003). Identification of a heparin-binding motif on adeno-associated virus type 2 capsids. J. Virol. *77*, 11072–11081.

37. Bishop, P.N. (2009). Vitreous as a substrate for vitreolysis. Dev. Ophthalmol. *44*, 7–19.

38. Gottlieb, J.L., Antoszyk, A.N., Hatchell, D.L., and Saloupis, P. (1990). The safety of intravitreal hyaluronidase. A clinical and histologic study. Invest. Ophthalmol. Vis. Sci. *31*, 2345–2352.

39. Staubach, F., Nober, V., and Janknecht, P. (2004). Enzyme-assisted vitrectomy in enucleated pig eyes: a comparison of hyaluronidase, chondroitinase, and plasmin. Curr. Eye Res. *29*, 261–268.

40. Kuppermann, B.D., Thomas, E.L., de Smet, M.D., and Grillone, L.R.; Vitrase for Vitreous Hemorrhage Study Groups (2005). Safety results of two phase III trials of an intravitreous injection of highly purified ovine hyaluronidase (Vitrase) for the management of vitreous hemorrhage. Am. J. Ophthalmol. *140*, 585–597.

41. Heegaard, S., Jensen, O.A., and Prause, J.U. (1986). Structure and composition of the inner limiting membrane of the retina. SEM on frozen resin-cracked and enzyme-digested retinas of Macaca mulatta. Graefes Arch. Clin. Exp. Ophthalmol. *224*, 355–360.

42. Cehajic-Kapetanovic, J., Eleftheriou, C., Allen, A.E., Milosavljevic, N., Pienaar, A., Bedford, R., Davis, K.E., Bishop, P.N., and Lucas, R.J. (2015). Restoration of Vision with Ectopic Expression of Human Rod Opsin. Curr. Biol. *25*, 2111–2122.

43. Kotterman, M.A., Yin, L., Strazzeri, J.M., Flannery, J.G., Merigan, W.H., and Schaffer, D.V. (2015). Antibody neutralization poses a barrier to intravitreal adeno-associated viral vector gene delivery to non-human primates. Gene Ther. *22*, 116–126.

44. Maclachlan, T.K., Lukason, M., Collins, M., Munger, R., Isenberger, E., Rogers, C., Malatos, S., Dufresne, E., Morris, J., Calcedo, R., et al. (2011). Preclinical safety evaluation of AAV2-sFLT01- a gene therapy for age-related macular degeneration. Mol. Ther. *19*, 326–334.

45. Ye, G.J., Budzynski, E., Sonnentag, P., Miller, P.E., Sharma, A.K., Ver Hoeve, J.N., Howard, K., Knop, D.R., Neuringer, M., McGill, T., et al. (2015). Safety and Biodistribution Evaluation in Cynomolgus Macaques of rAAV2tYF-CB-hRS1, a recombinant adeno-associated virus vector expressing retinoschisin. Hum. Gene Ther. Clin. Dev. *26*, 165–176.

46. Boye, S.E., Alexander, J.J., Witherspoon, C.D., Boye, S.L., Peterson, J.J., Clark, M.E., Sandefer, K.J., Girkin, C.A., Hauswirth, W.W., and Gamlin, P.D. (2016). Highly Efficient Delivery of Adeno-Associated Viral Vectors to the Primate Retina. Hum. Gene Ther. *27*, 580–597.

47. Enezi, Ja., Revell, V., Brown, T., Wynne, J., Schlangen, L., and Lucas, R. (2011). A "melanopic" spectral efficiency function predicts the sensitivity of melanopsin photoreceptors to polychromatic lights. J. Biol. Rhythms *26*, 314–323.

OMTM, Volume *9*

Supplemental Information

Efficacy and Safety of Glycosidic Enzymes for Improved Gene Delivery to the Retina following Intravitreal Injection in Mice

Jasmina Cehajic-Kapetanovic, Nina Milosavljevic, Robert A. Bedford, Robert J. Lucas, and Paul N. Bishop

Supplemental Material

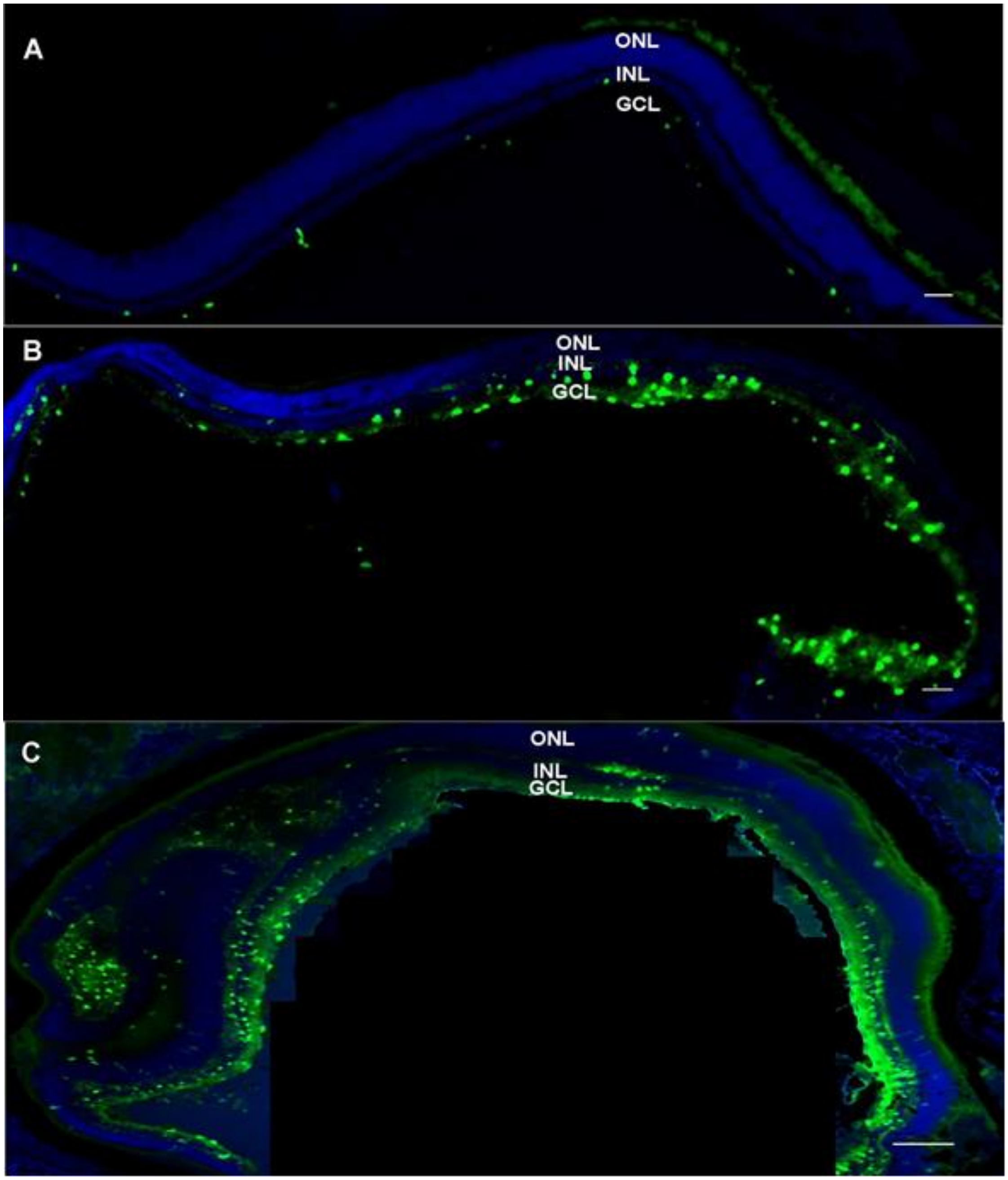

Supplemental Figure 1.

Transgene expression in *wild-type* retina using low dose AAV2-CAG-GFP in conjunction with glycosidic enzymes

(A-C) Exemplar images of transverse sections through a *wild-type* mouse retina >6 weeks after intravitreal delivery of AAV2-CAG-GFP ($2x10^8$ gc/eye) alone (A) or in conjunction with heparinase III (B) and hyaluronan lyase (C). Unamplified expression of GFP in cells of the ganglion cell layer (GCL) and inner nuclear layer (INL) is shown in green. Nuclei are counterstained with DAPI (blue). Calibration bar = 50µm.

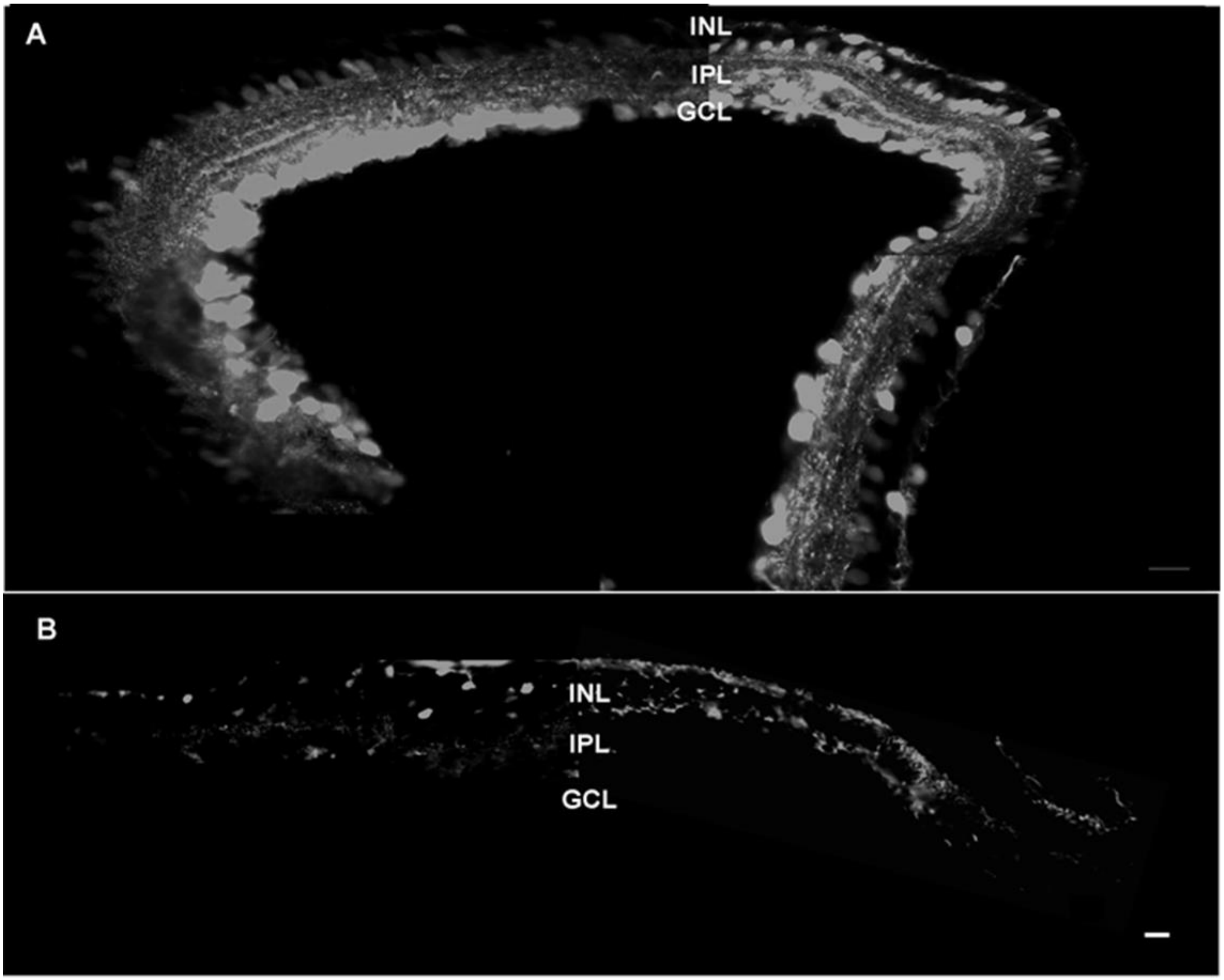

Supplemental Figure 2

Transgene expression in *rd*[1] retina using high dose AAV2-GFP in conjunction with glycosidic enzymes

(A, B) Montage of several high magnification images from an exemplar transverse section through an *rd*[1] mouse retina >6 weeks after intravitreal delivery of (A) AAV2- CAG-GFP (3 x10^{10} gc/eye) or (B) AAV2-grm6-GFP (3x10^{10} gc/eye) in conjunction with heparinase III and hyaluronan lyase combined. Expression of enhanced green fluorescent protein in cells of the ganglion cell layer (GCL) and inner nuclear layer (INL) is seen in (A) and in cells of the INL only in (B). Calibration bar = 50µm.
